# Endoscopic papillectomy of a rare isolated ampullary hamartoma

**DOI:** 10.1055/a-2325-2830

**Published:** 2024-06-12

**Authors:** Ahmed Altonbary, Fady Sabry, Hazem Hakim, Amr Elrabat, Wagdi Elkashef

**Affiliations:** 1 68779 Department of Gastroenterology and Hepatology, Mansoura University, Mansoura, Egypt; 2 68779 Department of Pathology, Mansoura University, Mansoura, Egypt

An isolated hamartoma in the ampulla of Vater is very rare, with only seven cases reported in the literature in patients without either Peutz–Jeghers or Cowdens syndrome as an underlying disease 1 . Herein, we report a rare case of isolated ampullary hamartoma diagnosed by endoscopic papillectomy.

A 69-year-old man was admitted to our hospital with recurrent abdominal pain and jaundice. Magnetic resonance cholangiopancreatography (MRCP) revealed a 2-cm ampullary mass, with dilatation of the common bile duct (CBD) to 12 mm and a normal pancreatic duct (PD). Laboratory investigations were unremarkable, apart from elevated serum bilirubin. Endoscopic ultrasound (EUS) evaluation showed a 2-cm hypoechoic mass with no intraductal extension or lymphadenopathy and intact muscularis propria.

Endoscopic papillectomy was performed as follows ( Video 1 ). After the lesion had been carefully assessed using the duodenoscope, the snare tip was anchored at the apex of the papilla then slowly opened and drawn down over the mass. The snare was then tightly closed without losing contact with the point of impaction above ( Fig. 1 **a** ). The entrapped mass was checked to ensure it was independently mobile from the duodenal wall before blend current was applied to resect the lesion en bloc. The resected specimen was retrieved by snaring and the resection bed was carefully examined ( Fig. 1 **b** ). The PD was then canulated, and this was followed by insertion of a pancreatic stent (5 cm, 5 Fr) ( Fig. 1 **c** ). No adverse events were reported during or after the procedure. Pathologic examination of the resected specimen revealed a polypoid lesion showing irregular glandular formations and strands of smooth muscle fibers, consistent with an ampullary hamartoma ( Fig. 2 ). Colonoscopy was then performed, which was clear up to the terminal ileum.

**Fig. 1:**
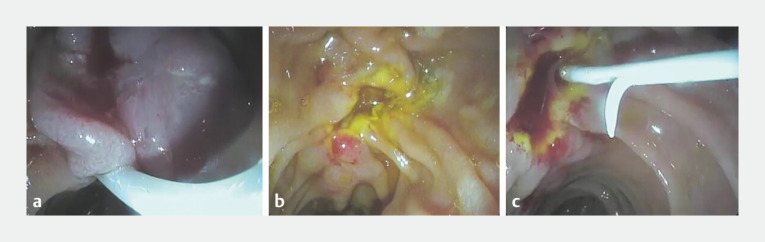
Endoscopic images during endoscopic papillectomy of an ampullary hamartoma showing: **a** a snare being tightly closed over the ampullary mass; **b** the clean resection bed; **c** a pancreatic stent in situ.

**Fig. 2:**
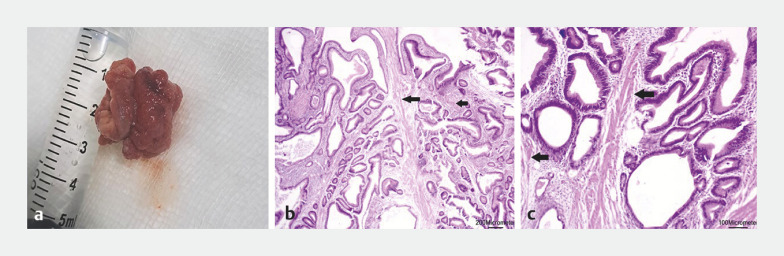
Pathologic examination of the retrieved specimen revealed: **a** on macroscopic view, a polypoid lesion; **b, c** the polypoid lesion with irregular glandular formations and strands of smooth muscle fibers (arrows) on a hematoxylin and eosin (H&E)-stained section, magnification: **b** × 40; **c** × 100.

Endoscopic papillectomy of an ampullary hamartoma.Video 1

Endoscopy_UCTN_Code_TTT_1AR_2AD

## Notes

Endoscopy E-Videos https://eref.thieme.de/e-videos
*E-Videos* is an open access online section of the journal *Endoscopy* , reporting on interesting cases and new techniques in gastroenterological endoscopy. All papers include a high-quality video and are published with a Creative Commons CC-BY license. Endoscopy E-Videos qualify for HINARI discounts and waivers and eligibility is automatically checked during the submission process. We grant 100% waivers to articles whose corresponding authors are based in Group A countries and 50% waivers to those who are based in Group B countries as classified by Research4Life (see: https://www.research4life.org/access/eligibility/ ). This section has its own submission website at https://mc.manuscriptcentral.com/e-videos .
